# Multiprofessional training for breastfeeding management in primary care in the UK

**DOI:** 10.1186/1746-4358-1-9

**Published:** 2006-04-28

**Authors:** Jennifer Ingram

**Affiliations:** 1 Centre for Child & Adolescent Health, Department of Community-based Medicine, University of Bristol, Hampton House, Cotham Hill, Bristol BS6 6JS, UK

## Abstract

**Background:**

Increasing breastfeeding initiation and duration rates is one of the UK Department of Health national targets for improving the health of the population. One reason contributing to the high rates of breastfeeding discontinuation may be that primary care teams may not have sufficient knowledge to help mothers overcome problems experienced in the early days and may also give conflicting advice. Previous studies have shown that general practitioners are happy to participate in practice-based educational sessions and have expressed a need for breastfeeding education. This study was carried out as part of the training to achieve 'UNICEF UK Baby Friendly Initiative in a community health care setting' status. It aimed to improve the breastfeeding expertise and advice about the management of breastfeeding problems within the primary care team using a CD-ROM breastfeeding learning package, and to assess the usefulness and acceptability of this educational intervention.

**Methods:**

Six UK general practitioner (GP) practices were involved in a questionnaire survey of multiprofessional groups before and after an interactive training session. This focussed on managing and solving problems, particularly mastitis and nipple thrush. The questionnaire included 20 questions on attitudes to and knowledge of breastfeeding, and eight multiple-choice questions on breastfeeding management. Non-parametric statistics (Mann-Whitney, Kruskal-Wallis and Wilcoxon tests) were used to compare the groups and to explore changes in knowledge after training.

**Results:**

Fifty primary care health professionals (29 GPs, 18 health visitors, 3 midwives) attended the sessions. There was an increase in scores relating to knowledge about breastfeeding after training, especially for the GPs and for those who did not have their own children. Health visitors improved their scores on recognition of the symptoms of poor attachment at the breast, and GPs showed greatest improvement in resolving sore nipples and recognising nipple thrush.

Changes in practice were reported and positive comments made about involving GPs and health visitors together in practice-based education.

**Conclusion:**

Using an electronic teaching resource is feasible for updating the knowledge of the primary care team. It can help to improve breastfeeding expertise and advice about breastfeeding problem management.

## Background

For many years UK government policy has been to increase rates of breastfeeding based on a strong evidence base of the health benefits conferred on mothers and children [1,2]. However, despite a series of initiatives by the Department of Health (DH) to promote breastfeeding, rates have increased only marginally in the past 20 years [3]. Increasing breastfeeding initiation and duration is one of the DH's national targets for improving the health of the population, as set out in the National Service Framework (NSF) for Children (Maternity)[4]. In the National Health Service (NHS) Priorities and Planning Framework 2003–6 Primary Care Trusts (PCTs) are tasked with increasing breastfeeding rates by 2% each year [5].

In 2000, the DH commissioned a review of the research evidence to examine practices which assist or inhibit the establishment of breastfeeding [6]. This review highlighted the scarcity of good well-designed research to inform practice, particularly on the prevalence of breastfeeding mismanagement by health professionals and the need to examine training programmes to educate caregivers in the essential skills of ensuring that women experience pain-free, effective feeding. It concluded that a co-ordinated approach to practice and research is needed, involving the full range of professionals who work with mothers and babies [6].

Factors which are known to be associated with successful breastfeeding include the correct positioning of the baby on the breast, a flexible approach to feed management, realistic expectations about breastfeeding and consistent advice between health professionals [6-8]. Reported reasons for giving up breastfeeding often revolve around physical factors such as perceived insufficient milk supply, sore and cracked nipples and confidence and satisfaction in breastfeeding [3]. One reason for the high rates of discontinuation in the UK may be that primary care teams may not have sufficient knowledge to help mothers overcome these problems and women may be given conflicting advice by different members of the team [6], A study in the US has reported that the main area that physicians needed more education was in breastfeeding problem solving [9]. Evidence suggests that the provision of extra support by professionals who have skills in breastfeeding will result in more mothers breastfeeding their babies for longer [8].

Little research has been published on issues relating to breastfeeding promotion and management in the primary care setting in the UK [10]. A Cochrane review of effective practice in the organisation of care (EPOC) has indicated that the most effective approach for modifying health professional behaviour and improving practice is educational outreach visits to the health centre [11]. Multiprofessional workshops are effective in improving collaboration within primary health care teams and those which include elements that are relevant, problem based, logical, challenging and interactive, and which build on experience are likely to be more successful [12].

A pilot study carried out by the author of the feasibility of delivering an evidence-based multiprofessional breastfeeding learning package to primary care teams concluded that general practitioners (GPs) were happy to participate in practice-based educational sessions and many expressed a need for breastfeeding education [13]. All health professionals involved in the study expressed a preference for training to be tailored to the needs of the practice. Breastfeeding rates for all the practices in the pilot improved in the six months after the training sessions had been delivered [13]. The package was modified as a result of the pilot study and the material transferred to a CD-ROM using Macromedia Flash software.

The objectives of the current study were to improve the breastfeeding expertise and the advice about the management of breastfeeding problems within the primary care team using a CD-ROM breastfeeding learning package and to explore the knowledge differences between health professional groups, gender and parity. Secondly the study aimed to assess the usefulness and acceptability of this educational intervention. The study was carried out as part of the training to achieve 'UNICEF UK Baby Friendly Initiative in a community health care setting' status [14], for which all the GPs who provided 'out-of-hours' cover at a Walk-In Centre in South Bristol were to be offered training in breastfeeding management and problem solving. This initiative and study was supported by the Bristol South & West PCT.

## Methods

During 2004, six practices (out of a possible 14 practices in South Bristol) were identified which had three or more doctors who provided 'out-of-hours' cover at the South Bristol Walk-In Centre. JI approached the practice managers and relevant health professionals in the practice to ascertain whether they would be willing to take part in breastfeeding training. The selection of practices was pragmatic, based on the amount of funding available (limited to six sessions) and a geographical spread across the PCT. Neighbouring practices were informed of the training, but only one GP came from a different practice. Lunchtime sessions for GPs and health visitors together were arranged with practice managers and the lead GPs and all health visitors were notified about the sessions. Some practices also invited community midwives and other nurses in the practice. Breastfeeding rates (any breastfeeding) in the area served by the Walk-In centre in South Bristol currently range from 20 to 35% at six weeks, which compares with the national rates of 42% of women who continue to breastfeed at six weeks post partum. The practices were in areas with young families of low socio-economic status, low household incomes, high unemployment and high levels of council house occupancy.

### Educational intervention

The intervention comprised a CD-ROM with accompanying information sheets on drugs for lactating women (taken from [15,16] produced by M Martindale, personal communication) and the treatment of nipple/breast thrush (candidiasis) (modified from [17]).

The CD-ROM [18] contains sections on:

• 'Why breastfeed' including the research based benefits with comprehensive references;

• 'How to breastfeed' which emphasises the importance of good positioning and attachment;

• 'Recognising and solving the problems'- both maternal and baby-related problems;

• 'Family support for breastfeeding'- the important role of the father and grandmothers;

• 'Antenatal preparation'- a section for midwives and health visitors.

The interactive session lasted about 40 minutes and concentrated on the section on managing and solving problems, particularly mastitis and thrush, since these clinical problems had been identified in the pilot study as being important for both groups. The participants also discussed management of these problems with each other and the facilitator (JI), by giving examples and responding to questions posed. All GPs and health visitors were given a copy of the CD-ROM and encouraged to try it out after the session to explore the sections not covered in detail at the session.

Before the session all attendees were asked to complete a questionnaire, which included 20 questions on attitudes to and knowledge of breastfeeding (see additional file: 1). The questions used Likert scale responses from strongly agree to strongly disagree. There were also eight multiple-choice questions on breastfeeding management covering the treatment of mastitis, breast milk insufficiency, attachment at the breast, sore nipples and nipple thrush. The questionnaire was based on a validated tool used by Scott et al with midwives in Scotland [19] and this shortened version took about ten minutes to complete. There was also a short section at the end of the questionnaire with demographic questions including age group, gender and whether they had children.

Four to six weeks after the training session, another questionnaire was sent to all those who had completed the first questionnaire, with a stamped addressed return envelope (see additional file: 2). This questionnaire included the knowledge and breastfeeding management questions from the initial questionnaire and further questions about the use of the CD-ROM and information sheets.

### Data analysis

The 13 attitude and seven knowledge questions on breastfeeding were each scored from one (low) to five (high), with a high score reflecting positive breastfeeding attitude and a high level of knowledge. These two groups were then summed to give total attitude and total knowledge scores, which could potentially range from 13 to 65 for attitude and 7 to 35 for knowledge. Histograms were plotted for each of the scores and most did not follow a normal curve suggesting that non-parametric methods of data analysis should be used for any hypothesis testing.

Data were analysed using SPSS v12 and p values of <0.05 were taken as the level of statistical significance. Spearman's correlations were calculated between the attitude and knowledge scores. Differences between genders, professions and participants with and without children were examined using Mann-Whitney and Kruskal-Wallis tests. Wilcoxon signed rank non-parametric tests were used to explore changes in total knowledge scores before and after training and in appropriate management of breastfeeding problems for individuals, using only data from those who completed both questionnaires.

Free text comments were coded and the frequencies of the comments reported.

Ethical permission for the study was given by the research ethics committee of United Bristol Healthcare Trust.

## Results

The training sessions took place from June to November 2004. Eighty health professionals (50 GPs, 25 health visitors, 5 midwives) were invited to attend the practice-based breastfeeding sessions. Six sessions were delivered, at which 29 GPs, 18 health visitors and 3 midwives completed the initial questionnaires (total 50; 63% of those invited). In one health centre some practice nurses also attended out of interest in the topic, but they were not asked to complete questionnaires as they did not advise breastfeeding women. Thirty-six replies (72%) were received to the second questionnaire, sent four to six weeks after each training session, from 21 GPs, 13 health visitors and 2 midwives.

There were 40 female and 10 male health professionals at the training sessions, 39 of whom had children. All but one of these children had been breastfed. Table 1 shows the characteristics by professional group.

**Table 1:** Characteristics of the sample by health professional group

	**n**	**Gender female:male**	**Have children**
GP	29	19:10	21
Health visitor	18	18:0	15
Midwife	3	3:0	3

Before the training session, the mean attitude score was 55.3 (SD 5.4) with a range of 38 to 65, indicating very positive attitudes towards breastfeeding in these groups. The mean knowledge score was 28.4 (SD 4.3) with a range of 19 to 35, again reflecting the high knowledge levels of those who attended. After training the mean knowledge score was 29.7 (SD 3.6) for the 36 who returned this questionnaire, but this overall increase was not statistically significant (Wilcoxon p = 0.23). There was a significant positive correlation between the knowledge and attitude scores before training (Spearman r = 0.71, p < 0.001), indicating that more knowledgeable health professionals also had more positive breastfeeding attitudes. Table 2 shows the mean scores for attitude and knowledge for the different professional groups before and after the training session. Comparisons were made for those individuals who completed both pre and post intervention questionnaires. There were significant differences between the groups before and after training, particularly in breastfeeding knowledge, with GPs having the lowest scores and midwives the highest (p < 0.001). The mean knowledge scores for GPs increased after training but the increase was not statistically significant, probably because they started at quite a high level (Wilcoxon p = 0.18).

**Table 2:** Mean scores for breastfeeding attitude and knowledge before and after training

**Staff group**		**Attitude before **(max* = 65)		**Knowledge before **(max* = 35)			**Knowledge after **(max* = 35)	
	n	mean	range	mean	range	n	mean	range

**GPs**	29	53.7	38–65	26.4	19–33	21	28.2	23–34
**Health visitors**	18	57.2	50–63	30.7	24–35	13	31.4	25–35
**Midwives**	3	59.7	58–63	34.3	33–35	2	34.5	34–35
Kruskal-Wallis test		Chi-square 7.34	p=0.025	Chi-square 16.89	p < 0.001		Chi-square 9.79	p=0.007

*max = maximum score

Table 3 shows that there were no significant differences between males and females in their attitude towards breastfeeding or knowledge. However those who had children showed significantly more positive attitudes towards breastfeeding and also had significantly higher knowledge scores before training. This difference was not apparent after training due to an increase in knowledge scores for those without children, which was statistically significant (Wilcoxon p = 0.04).

**Table 3:** Comparison between male and female health professionals, and those with and without children for attitude and knowledge scores before and after training

**Group**		**Attitude before **(max* = 65)		**Knowledge before **(max* = 35)			**Knowledge after **(max* = 35)	
	n	mean	range	mean	range	n	mean	range

**Male**	10	56.3	50–65	28.2	25–33	7	28.0	23–33
**Female**	40	55.1	38–63	28.5	19–35	29	30.1	24–35
Mann-Whitney		192.0	p=0.817	204.5	p=0.715		69.0	p=0.191
**With children**	39	56.7	50–65	29.3	22–35	28	30.2	23–35
**Without children**	11	50.3	38–62	25.6	19–34	8	28.1	24–34
Mann-Whitney		98.0	p=0.002	131.5	p=0.019		72.0	p=0.126

*max = maximum score

The main changes seen in the management of breastfeeding problems were significant increases in appropriate advice to women with mastitis to keep breastfeeding on both breasts, greater recognition of the symptoms of poor attachment at the breast, greater knowledge of how to resolve sore nipples, and increased recognition of the symptoms of nipple thrush as shown in Table 4. Health visitors particularly improved their scores on recognition of the symptoms of poor attachment at the breast, and GPs showed greatest improvement in resolving sore nipples and recognising nipple thrush.

**Table 4:** Changes in scores for management of breastfeeding problems after training

**Breastfeeding management problem**	**Mean Score before training**	**Mean Score after training**	**Wilcoxon ** **p value**
Appropriate advice for mastitis (Continue to feed on both breasts)	89% appropriate	100% appropriate	2.0 0.046
Appropriate advice for breast milk insufficiency (Increase breastfeeding frequency and seek expert help) (max score = 3)	2.6	2.5	1.0 0.317
Correct symptoms to indicate a poorly attached baby (very frequent feeding, mother has sore nipples/repeated engorgement/mastitis).(max score = 4)	3.1	3.7	3.12 0.002
Resolution of sore nipples (check for nipple thrush, apply breast milk, seek expert help with attachment, apply lanolin to cracked nipples).(max score = 4)	2.5	3.0	3.35 0.001
Correct symptoms of nipple thrush (pink, sensitive, tender, cracked nipples; shooting, burning pains in the breast).(max score = 3)	2.2	2.7	2.98 0.003

The follow-up questionnaire showed that since the session, 12 health professionals (33%) (7 GPs, 4 HVs, 1 MW) had used the CD-ROM and 17 (47%) the information sheets, but the others reported that they had not had an opportunity to use them yet. All had found them useful for their practice. Changes in practice were reported by 15 (42%) and these included advice about thrush (n = 7, 46%), about sore nipples (n = 3, 20%), mastitis (n = 3, 20%), and more focus on positioning and attachment as a cause of problems (n = 3, 20%).

Qualitative comments (made by 16 participants) about the session were very positive and most (n = 12, 75%) had found it informative, very useful, helpful and appropriate. Others commented that "*it was good to have GPs and health visitors discussing these topics together at a practice-based session*" and that "*it should form part of mandatory training for health professionals*".

## Discussion

This evaluation has shown that a short interactive multiprofessional breastfeeding session for primary care teams can enhance the knowledge of GPs and health visitors. Almost half of the participants subsequently used the resources within two months to reinforce their knowledge and most found the content and presentation very informative.

Others who have used interactive multimedia interventions [20] for breastfeeding education or internet based teaching programmes [21] have found these new models of education to be well accepted by clinicians. Hillenbrand and Larsen [20] showed that by using role-play, video and discussions, paediatric residents' breastfeeding knowledge, confidence and clinical behaviour was enhanced, and the areas which showed significant improvement included breast milk insufficiency, mastitis treatment and drugs for lactating mothers. In the current study inconsistencies in breastfeeding management by health professionals were linked to poor understanding of the treatment of more complex breastfeeding situations, including nipple thrush, the ramifications of poor infant attachment to the breast and the resolution of sore nipples. Knowledge of these problems and reported practice improved after the training session and associated discussion.

It is often assumed that training health professionals reduces variation in clinical practice and leads to improved patient outcomes, which will only be true if the training improves knowledge and skills [21]. Using a validated tool to assess knowledge and skills makes assessment of participants across professional groups more reliable. Our study was not designed to measure long-term effects on knowledge or behavioural change, but the post session questionnaire did demonstrate retention of short-term information, to be reinforced when necessary with the use of a CD-ROM.

Scott et al. showed by using a reliable and valid measurement scale for evaluating breastfeeding attitudes, knowledge and management practices of health professionals, that there is a positive correlation between attitude and knowledge scores, and that midwives with personal breastfeeding experience had higher attitude scores than those without [19]. These findings were confirmed by the current study with GPs, health visitors and community midwives, using a shorter form of the validated tool.

Limitations of the study include the small numbers involved and the pre and post intervention design of the evaluation, which can only report statistical associations, and the lack of a control group. During the study period there were no other policy or practice changes which could have influenced the participants' knowledge. Delivering this type of practice-based session, with associated resources, was appreciated by the primary care teams, even though it was rather time-consuming for the facilitator to visit individual practices. The costs of the facilitator, who is an expert in lactation physiology and management, were covered by the PCT, and she is also a resource used locally by health professionals. This may not be possible or feasible in other PCTs, but it is hoped that most health professionals should be able to use the CD-ROM as a stand alone package.

Over 60% of the health professionals, who were notified about the session by practice managers, and invited to attend, came to the training and those who did not attend were reported by their colleagues to be those who probably rarely advised breastfeeding women. It was inevitable that those who attended were positive about breastfeeding, but the evaluation showed that they still had capacity to increase their knowledge about the management of breastfeeding problems, particularly GPs and those without their own children. A recommendation from this study for future research would include a larger evaluation which also explored the training in terms of women's experiences of care and measured breastfeeding outcomes.

Training primary care health professionals in improved breastfeeding management is an important part of a multi-faceted approach towards improving breastfeeding rates, as advocated by the UNICEF UK Baby Friendly Initiative [14]. Ingram and Johnson [22] have also reported that the opportunistic discussion about the benefits and management of breastfeeding with mothers and other family members can help to improve breastfeeding continuation rates.

GPs have had a declining role in maternity and postnatal care in Britain over recent years and maternity care is midwife-led in many areas. In the UK, midwifery care continues for ten to 14 days postpartum and then passes to health visitors, who are often only able to visit families with high health needs. However many breastfeeding problems arise after mothers have been discharged from midwifery care and they turn to health visitors or their GP for advice and treatment. It is therefore important to have an up to date and accessible resource for health professionals, who may not come across breastfeeding problems very often, to enable them to give physiologically accurate and evidence-based advice.

## Conclusion

Using an electronic teaching resource in a practice-based training session is feasible for updating the knowledge of a primary care team and the participants found it to be acceptable. It can help to improve breastfeeding expertise, may improve the consistency of advice about the management of breastfeeding problems, and thus help to improve breastfeeding continuation rates in the community.

## Competing interests

The CD is available from Dr Jenny Ingram jenny.ingram@bristol.ac.uk(price £8).

## Supplementary Material

Additional file 1 Breastfeeding Questionnaire 1 Click here for file

Additional file 2 Breastfeeding Questionnaire 2 Click here for file
