# Supplementary material for: Multiprofessional training for breastfeeding management in primary care in the UK
Source: Int Breastfeed J. 2006 Apr 28;1:9. doi: 10.1186/1746-4358-1-9 (PMC1475559; doi:10.1186/1746-4358-1-9)
Supplement: Additional file 1 — Breastfeeding Questionnaire 1 [file 1746-4358-1-9-S1.doc]

# BREASTFEEDING QUESTIONNAIRE 1

For each statement below, please indicate how much you agree or disagree by circling the number that most closely corresponds to your opinion.

# Section 1. Strongly Strongly

disagree agree

| 1. Health professionals should actively encourage all mothers in their practices to try breastfeeding | | 1 | | 2 | 3 | | | 4 | 5 |
| --- | --- | --- | --- | --- | --- | --- | --- | --- | --- |
| 1. Formula feeding is good way of letting fathers care for the baby | | 1 | | 2 | 3 | | | 4 | 5 |
| 1. Breast milk is the ideal food for babies | | 1 | | 2 | 3 | | | 4 | 5 |
| 1. A mother who occasionally drinks alcohol should not breastfeed her baby | | 1 | | 2 | 3 | | | 4 | 5 |
| 1. Mothers intending to breastfeed should expect sore nipples as a normal part of breastfeeding | | 1 | | 2 | 3 | | | 4 | 5 |
| 1. Health professionals have little influence on a woman’s decision to continue breastfeeding | | 1 | | 2 | 3 | | | 4 | 5 |
| 1. A breastfed baby is likely to have fewer infections than a formula fed baby | | 1 | | 2 | 3 | | | 4 | 5 |
| 1. Formula fed babies are more likely to be overfed than breastfed babies | | 1 | | 2 | 3 | | | 4 | 5 |
| 1. Breastfeeding is beneficial to a mother’s health | | 1 | | 2 | 3 | | | 4 | 5 |
| 1. Breast milk alone can satisfy most babies for approximately the first six months | | 1 | | 2 | 3 | | | 4 | 5 |
| 1. Formula milk is more easily digested than breast milk | 1 | | 2 | | | 3 | 4 | | 5 |
| 1. Breastfeeding provides health benefits for infants that cannot be provided by formula | 1 | | 2 | | | 3 | 4 | | 5 |
| 1. Fathers feel left out if a mother breastfeeds | 1 | | 2 | | | 3 | 4 | | 5 |

**Section 2. Strongly Strongly**

disagree agree

| 1. A woman who is fully breastfeeding is less likely to become pregnant three months after delivery than a woman who is formula feeding | 1 | 2 | 3 | 4 | 5 |
| --- | --- | --- | --- | --- | --- |
| 1. Supplemental feeding is detrimental to the establishment of a good milk supply | 1 | 2 | 3 | 4 | 5 |
| 1. It is usually advisable for babies to receive a formula feed before the first breastfeed | 1 | 2 | 3 | 4 | 5 |
| 1. Frequent breastfeeding in the early newborn period can help reduce jaundice | 1 | 2 | 3 | 4 | 5 |
| 1. Growth patterns of breastfed infants differ from those of formula fed infants | 1 | 2 | 3 | 4 | 5 |
| 1. If a breastfed infant has not regained his birth weight by two weeks of age, the mother should be encouraged to begin supplementing with formula | 1 | 2 | 3 | 4 | 5 |
| 1. A mother of an infant who feels she has insufficient milk should “top up” with a bottle after each feed | 1 | 2 | 3 | 4 | 5 |

1. **If a woman develops mastitis, what do you usually advise her to do about breastfeeding?** (*Circle* ***ANY*** *that apply*)

Continue to feed on both sides 1

Stop feeding on the affected side 2

Stop feeding altogether 3

Prescribe antibiotics…………………………………………………………………………..4

Unsure / Don’t know 5

1. **If a mother complains of breast milk insufficiency which of the following options will help to resolve the problem:** (*Circle* ***ANY*** *that apply*)

Increase frequency of breast milk feedings 1

Top up each breastfeed with a bottle of formula 2

Seek expert assistance with positioning and attachment 3

Advise mother to drink more fluids 4

Unsure / Don’t know 5

1. **Which of the following symptoms could indicate POOR attachment at the breast**: (*Circle* ***ANY*** *that apply*)

Baby feeds very frequently and is unsettled 1

Mother has sore and cracked nipples 2

Mother reports repeated engorgement 3

Mother has mastitis 4

Unsure / Don’t know 5

1. **If a mother complains of sore nipples which of the following options will**

**help to resolve the problem:** *(Circle* ***ANY*** *that apply)*

Stop feeding on the affected side ……………………….………………………………… 1

Check for symptoms of nipple thrush ………………………………………….………... 2

Advise mother to apply breast milk to nipples ……………………………………..……. 3

Seek expert assistance with positioning and attachment ………….…………………… 4

Advise mother to apply lanolin to nipples ………………………………………………… 5

Unsure / Don’t know ……………………………………………………………………….. 6

**13. The symptoms of nipple thrush can include:** *(Circle* ***ANY*** *that apply)*

Nipples are pink, sensitive and tender ………………………………………………….. 1

Nipples are cracked …………………………………………………………………...….. 2

Shooting, burning pains in the breast …………………………………………………… 3

Breast is lumpy and red ……………………………………………………………….. 4

White patches on nipple or breast… ………………. …………………………………. 5

Unsure / Don’t know………………………………………………………………………. 6

**Section 3.**

1. Are you MALE ❑ or FEMALE ❑ ?

2. Do you have any children? Yes ❑ No ❑

**number**

3. If **YES,**

1. How many were breastfed (including mixed feeding)?
2. How many were formula fed exclusively?

***Thank you for taking the time to complete this questionnaire.***
