# Supplementary material for: Multiprofessional training for breastfeeding management in primary care in the UK
Source: Int Breastfeed J. 2006 Apr 28;1:9. doi: 10.1186/1746-4358-1-9 (PMC1475559; doi:10.1186/1746-4358-1-9)
Supplement: Additional file 2 — Breastfeeding Questionnaire 2 [file 1746-4358-1-9-S2.doc]

# BREASTFEEDING QUESTIONNAIRE 2

As a follow-up to the recent training session that you attended, we would be grateful if you would complete the following questionnaire about your current advice to breastfeeding mothers.

For each statement below, please indicate how much you agree or disagree by circling the number that most closely corresponds to your opinion.

**Strongly Strongly**

disagree agree

| 1. A woman who is fully breastfeeding is less likely to become pregnant three months after delivery than a woman who is formula feeding | 1 | 2 | 3 | 4 | 5 |
| --- | --- | --- | --- | --- | --- |
| 1. Supplemental feeding is detrimental to the establishment of a good milk supply | 1 | 2 | 3 | 4 | 5 |
| 1. It is usually advisable for babies to receive a formula feed before the first breastfeed | 1 | 2 | 3 | 4 | 5 |
| 1. Frequent breastfeeding in the early newborn period can help reduce jaundice | 1 | 2 | 3 | 4 | 5 |
| 1. Growth patterns of breastfed infants differ from those of formula fed infants | 1 | 2 | 3 | 4 | 5 |
| 1. If a breastfed infant has not regained his birth weight by two weeks of age, the mother should be encouraged to begin supplementing with formula | 1 | 2 | 3 | 4 | 5 |
| 1. A mother of an infant who feels she has insufficient milk should “top up” with a bottle after each feed | 1 | 2 | 3 | 4 | 5 |

1. **If a woman develops mastitis, what do you usually advise her to do about breastfeeding?** (*Circle* ***ANY*** *that apply*)

Continue to feed on both sides 1

Stop feeding on the affected side 2

Stop feeding altogether 3

Prescribe antibiotics…………………………………………………………………………..4

Unsure / Don’t know 5

1. **If a mother complains of breast milk insufficiency which of the following options will help to resolve the problem:** (*Circle* ***ANY*** *that apply*)

Increase frequency of breast milk feedings 1

Top up each breastfeed with a bottle of formula 2

Seek expert assistance with positioning and attachment 3

Advise mother to drink more fluids 4

Unsure / Don’t know 5

1. **Which of the following symptoms could indicate poor attachment at the breast**: (*Circle* ***ANY*** *that apply*)

Baby feeds very frequently and is unsettled 1

Mother has sore and cracked nipples 2

Mother reports repeated engorgement 3

Mother has mastitis 4

Unsure / Don’t know 5

1. **If a mother complains of sore nipples which of the following options will**

**help to resolve the problem:** *(Circle* ***ANY*** *that apply)*

Stop feeding on the affected side ……………………….………………………………… 1

Check for symptoms of nipple thrush ………………………………………….………... 2

Advise mother to apply breast milk to nipples ……………………………………..……. 3

Seek expert assistance with positioning and attachment ………….…………………… 4

Advise mother to apply lanolin to nipples ………………………………………………… 5

Unsure / Don’t know ……………………………………………………………………….. 6

**13. The symptoms of nipple thrush can include:** *(Circle* ***ANY*** *that apply)*

Nipples are pink, sensitive and tender ………………………………………………….. 1

Nipples are cracked …………………………………………………………………...….. 2

Shooting, burning pains in the breast …………………………………………………… 3

Breast is lumpy and red ……………………………………………………………….. 4

White patches on nipple or breast… ………………. …………………………………. 5

Unsure / Don’t know………………………………………………………………………. 6

**14.** Have you **changed any advice** that you give to

breastfeeding mothers since attending the session? Yes ❑ No ❑

If **YES,** which advice is different? ……………………………………………………..

…………………………………………………………………………………………….

**15**. Have you used any of the following and do you find them useful?

Used it Useful

yes no yes no

a) The Breastfeeding Package CD ❑ ❑ ❑ ❑

b) Drugs for breastfeeding women ❑ ❑ ❑ ❑

c) Thrush and breastfeeding sheet ❑ ❑ ❑ ❑

**16**. Do you have any comments about the breastfeeding management training session? .

…………………………………………………………………………………………………….

…………………………………………………………………………………………………….

***Thank you for taking the time to complete this questionnaire.***

Please return to: Dr Jenny Ingram

Centre for Child and Adolescent Health,

Hampton House, Cotham Hill,

Bristol BS6 6JS
